# A Label-Free, Mix-and-Detect ssDNA-Binding Assay Based on Cationic Conjugated Polymers

**DOI:** 10.3390/bios13010122

**Published:** 2023-01-10

**Authors:** Pengbo Zhang, Mohamad Zandieh, Yuzhe Ding, Lyuyuan Wu, Xiaoyu Wang, Juewen Liu, Zhengping Li

**Affiliations:** 1 School of Chemistry and Biological Engineering, University of Science and Technology Beijing, 30 Xueyuan Road, Haidian District, Beijing 100083, China; 2 Department of Chemistry, Waterloo Institute for Nanotechnology, University of Waterloo, 200 University Avenue West, Waterloo, ON N2L 3G1, Canada; 3 School of Materials Science and Engineering, University of Science and Technology Beijing, 30 Xueyuan Road, Haidian District, Beijing 100083, China

**Keywords:** cationic conjugated polymer, fluorescence, label-free, ssDNA, biosensor

## Abstract

The accurate, simple, and efficient measurement of the concentration of single-stranded DNA (ssDNA) is important for many analytical applications, such as DNA adsorption, biosensor design, and disease diagnosis, but it is still a challenge. Herein, we studied a cationic conjugated polymer (CCP)-based ssDNA assay taking advantage of the obvious fluorescence change of CCPs upon binding ssDNA. Poly(3-(3′-N,N,N-triethylamino-1′-propyloxy)-4-methyl-2,5-thiophene hydrochloride) (PMNT) achieved an apparent dissociation constant (*K*_d_) of 57 ± 4 nM for ssDNA, indicating a very high binding affinity between PMNT and ssDNA. This allowed us to develop a CCP-based ssDNA biosensor with a detection limit of 0.6 nM, similar to the fluorescence-dye-based method using SYBR Green I and SYBR Gold. Our CCP-based biosensor produced smaller differences among ssDNA samples with different base compositions. In addition, the existence of double-stranded DNA (dsDNA) at different concentrations did not interfere with the fluorescence of PMNT, indicating that our CCP-based biosensor was more suitable for the measurement of ssDNA. Compared with fluorescence-intensity-based quantification, our CCP system allowed ratiometric quantification, which made the calibration easier and more robust. We then applied our method to the quantification of ssDNA on AuNPs using both unmodified and thiolated ssDNA, and the accurate quantification of ssDNA was achieved without any fluorophore modification. This method provides an alternative approach for the measurement of ssDNA.

## 1. Introduction

The highly sensitive and specific detection of single-stranded nucleic acids is important in clinical diagnostics and medical research. Almost all primers, DNA aptamers, and DNAzymes are single-stranded oligonucleotides. Compared with the remarkable development of dyes for staining double-stranded DNA (dsDNA), single-stranded DNA (ssDNA) quantification strategies have been far less explored.

As a standard method for nucleic acid detection, the polymerase chain reaction (PCR)-based technique has been widely used for single-stranded RNA and ssDNA detection [1,2,3]. Although very high sensitivity and accuracy have been achieved, PCR-based methods still suffer from tedious labels, high costs, and the need for precise thermal cycle devices, with limited application in low-resource settings. The direct fluorescence-based method is easier and more cost effective. Some nucleic acid binding dyes, such as SYBR Green I (SGI) and SYBR Gold, are reported to show dramatically enhanced fluorescence after binding with dsDNA and ssDNA, respectively [4,5,6]. Most of these dyes are more likely to intercalate into the groove of dsDNA; thus, a high concentration of dsDNA may interfere with the detection of ssDNA. Colorimetric assays provide an alternative means of ssDNA detection [7,8]. Gold nanoparticles (AuNPs) with the unique localized surface plasmon resonance effect show obvious color changes upon crosslinking with ssDNA [9,10,11], but this method relies on covalent modified AuNPs with expensive thiolated ssDNA. ssDNA and dsDNA have different binding affinities toward unmodified AuNPs. As a result, ssDNA can better protect AuNPs from salt-induced aggregation [12,13,14,15]. This mechanism has been utilized for the quick detection of ssDNA, but this effect is dependent on the ssDNA length and reaction buffer [16,17]. The discovery of novel pathways to develop biosensors for ssDNA measurement with simple instrumentation and an easy preparation procedure is still a challenging issue.

Cationic conjugated polymers (CCPs) offer extraordinary possibilities for the development of highly sensitive and specific DNA biosensors due to their molecular wire effect and strong light-harvesting ability compared with small molecular analogs [18,19,20]. The fluorescence resonance energy transfer (FRET) between CCPs and fluorescence dye or fluorophore labeled on ssDNA enables obvious optical amplification [21,22,23]. In addition, CCPs, especially polythiophene derivatives (PTs), show obvious conformational changes upon interacting with ssDNA and dsDNA [24,25,26]. The binding of ssDNA leads to the aggregation of the conjugated backbone of PTs and the formation of a planar conformation, resulting in a color change and a significantly decreased or red-shifted fluorescence. Upon the addition of complementary DNA (cDNA), ssDNA binds with cDNA perfectly instead of PTs, leading to the significant recovery of color and fluorescence [27,28]. This makes PTs promising for developing DNA hybridization and cDNA detection sensors. In consideration of the ssDNA nature of aptamers and the obvious conformational changes before and after binding with targets, the absorption and fluorescence changes of PTs have also been utilized for the detection of metal ions, small targets, and proteins [29,30,31,32].

In this paper, we investigated the binding affinity between CCPs and ssDNA, utilizing the fluorescence changes of CCPs in response to conformational changes after binding with ssDNA. We first optimized the concentration of salt and CCPs and assessed the effect of the pH and DNA sequence on the fluorescence changes of CCPs. We also studied the influence of dsDNA, which might interfere with the measurement of ssDNA. Two commercial fluorescence dyes, SGI and SYBR Gold, were utilized to verify the results of our CCP-based ssDNA-binding assay. Finally, we applied our CCP-based assay to the measurement of ssDNA on AuNPs and realized the accurate quantification of ssDNA without any fluorophore label.

## 2. Materials and Methods

### 2.1. Chemicals

The DNA used in this work was ordered from Integrated DNA Technologies (Coralville, IA, USA). The sequences of the DNA are shown in Table S1. SGI and SYBR Gold were purchased from Sigma-Aldrich. Sodium chloride (NaCl) and 4-(2-hydroxyethyl) piperazine-1-ethanesulfonic acid (HEPES) were obtained from Mandel Scientific (Guelph, ON, Canada). Poly(3-(3′-N,N,N-triethylamino-1′-propyloxy)-4-methyl-2,5-thiophene hydrochloride) (PMNT) was ordered from Okeanos Tech. Co., Ltd. and prepared according to the method described in previous reports [27,33]. All the buffers and solutions were prepared using Milli-Q water.

### 2.2. CCP-Based Binding Assay

For the binding of PMNT with DNA, 5 μM PMNT (5 μM monomer unit or 37.9 nM polymer concentration) was added to the reaction buffer, which contained 20 mM HEPES (pH 7.5) and 0.3 M NaCl. Then, different concentrations of ssDNA or dsDNA were added and mixed. The reaction mixture with a final volume of 100 μL was immediately placed in a 96-well plate and subjected to the detection process under an excitation of 392 nm using a microplate reader (Tecan Spark). We then monitored the fluorescence signal of PMNT from 450 nm to 650 nm to draw the fluorescence spectra. The fluorescence ratio was defined as the peak intensity ratio of 580 nm to 530 nm. The dissociation constant (*K*_d_) value was calculated using the equation ∆F = F_max_[*C*]/(*K*_d_ + [*C*]) [34,35], where ∆F stands for the fluorescence ratio change of PMNT, defined as ∆F = F_580_/F_530_(ssDNA)-F_580_/F_530_(Blank); [*C*] is the ssDNA concentration; and F_max_ represents the maximal fluorescence ratio change upon saturated binding.

### 2.3. SGI and SYBR Gold-Based Fluorescence Assay

The SGI and SYBR Gold-based fluorescence assay used the same reaction buffer as the CCP-based binding assay (20 mM HEPES (pH 7.5) and 0.3 M NaCl). The final concentration of SGI or SYBR Gold was 0.5×. Different concentrations of ssDNA or dsDNA were added to a mixture containing SGI or SYBR Gold to a final volume of 100 μL. After incubating for 2 min, the fluorescence of the SGI and SYBR Gold was detected under an excitation wavelength of 470 nm. The emission fluorescence signal was recorded from 515 to 600 nm.

### 2.4. Quantification of Unlabeled ssDNA on AuNPs

Firstly, 100 nM unlabeled ssDNA and 2 nM AuNPs (13 nm) were added to 10 mM phosphate buffer (PB) (pH 7.5) with 50 mM NaCl. The reaction mixture was mixed and incubated at room temperature for 10 min. To remove the unbound ssDNA, the mixture was centrifuged at 15,000 rpm for 15 min and washed three times with PB (10 mM). To quantify the absorbed ssDNA, 100 μL KCN (10 mM) was added to totally dissolve the AuNP core. The released ssDNA (25 μL) was mixed with 5 μM PMNT in 20 mM HEPES (pH 7.5) with 0.3 M NaCl. The mixture was immediately added to the 96-well plate and subjected to the detection procedure under an excitation wavelength of 392 nm. The fluorescence ratio of 580 nm to 530 nm was utilized to quantify the concentration of absorbed ssDNA. Finally, the number of DNA strands attached to each AuNP was calculated.

For fluorescence-based detection, 100 nM FAM-labeled ssDNA was used. The released FAM-labeled ssDNA was then detected by measuring the fluorescence signal.

### 2.5. Quantification of Thiolated ssDNA on AuNPs

We first prepared the spherical nucleic acids (SNAs) using the freezing-directed method [36]. Briefly, 3 μM 5′-SH-labeled ssDNA and 10 nM AuNPs (13 nm) were mixed and placed at −20 °C for 3 h. The mixture was then taken out and placed at room temperature to slowly thaw. In order to remove the unbound ssDNA, the mixture was centrifuged at 15,000 rpm for 15 min and washed three times with PB (10 mM). To quantitively measure the exact number of ssDNA strands on each AuNP, 200 μL KCN (10 mM) was added to totally dissolve the AuNP core. The released ssDNA (2 μL) was mixed with 5 μM PMNT in 20 mM HEPES (pH 7.5) with 0.3 M NaCl. The mixture was immediately added to the 96-well plate and subjected to the detection procedure under an excitation wavelength of 392 nm. The fluorescence ratio of 580 nm to 530 nm was utilized to quantify the concentration of absorbed ssDNA. Finally, the number of DNA strands attached to each AuNP was calculated.

For fluorescence-based detection, 3 μM dual-labeled ssDNA (5′-SH-DNA-FAM-3′) was used. The released dual-labeled ssDNA (50 μL) was added into 20 mM HEPES (pH 7.5) with 0.3 M NaCl and then detected by measuring the fluorescence signal and quantified using the standard curve.

## 3. Results

### 3.1. The Mechanism of Our CCP-Based ssDNA-Binding Assay

**Figure 1:**
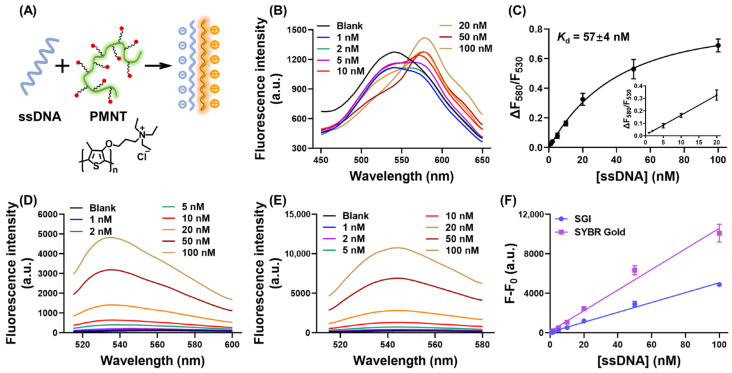
(**A**) Schematic representation of the CCP-based assay for ssDNA measurement. (**B**) The fluorescence spectra of PMNT upon adding different concentrations of ssDNA. The concentration of PMNT was 5 μM, as calculated by repeat units. (**C**) The fluorescence ratio changes of PMNT in response to ssDNA concentration. Inset: the linear response from 1 to 20 nM ssDNA. The fluorescence spectra of SGI (**D**) and SYBR Gold (**E**) after binding with different concentrations of ssDNA. The concentration of SGI and SYBR Gold was 0.5×. (**F**) The fluorescence intensity changes of SGI and SYBR Gold in response to ssDNA concentration.

A cationic polythiophene derivate, PMNT, was used as the model CCP. The chemical structure is depicted in Figure 1A. As a conjugated polymer with 132 thiophene monomer units, the average molecular weight of PMNT was about 38 kDa (with a molecular weight distribution of 1.87) [31]. In the free conformation, PMNT showed a randomly coiled nonplanar conformation. The initial fluorescence spectrum of PMNT had a single peak at the wavelength of 530 nm. The main binding force between PMNT and ssDNA comprised electrostatic and hydrophobic interactions, which caused PMNT to show a highly conjugated and planar conformation, resulting in decreased or red-shifted fluorescence. As depicted in Figure 1B, when adding 1 nM ssDNA, the fluorescence of PMNT only showed a decreased effect at 530 nm. After adding 2 nM ssDNA, PMNT showed a slightly red-shifted fluorescence at 580 nm together with decreased fluorescence at 530 nm. Further increasing the ssDNA concentration caused the red-shifted fluorescence at 580 nm to become obvious. For the quantitative measurement of ssDNA, a fluorescence ratio of 580 nm to 530 nm was utilized, and the fluorescence ratio change was defined as ∆F_580_/F_530_ = F_580_/F_530_(ssDNA)-F_580_/F_530_(Blank). Although the fluorescence for 1 nM and 2 nM ssDNA at 530 nm was slightly lower than that for 5 nM and 10 nM ssDNA, the fluorescence at 580 nm for 1 nM and 2 nM ssDNA was much lower than that for 5 nM and 10 nM ssDNA, resulting in a relatively low fluorescence ratio, as shown in Figure 1C. We then studied the relationship between ∆F_580_/F_530_ and ssDNA concentration. Figure 1C shows a standard binding curve with a *K*_d_ of 57 ± 4 nM. A fine linear relationship was achieved in the range of 1 to 20 nM ssDNA with a detection limit of 0.6 nM. The use of ratiometric measurements would have made the calibration more robust, since a slight change in lamp intensity or detector sensitivity is unlikely to significantly change the ratio. In contrast, the absolute fluorescence intensity may be strongly affected.

For comparison, two commercial dyes, SGI and SYBR Gold, were selected for label-free ssDNA measurement. Although known for dsDNA staining, SGI was reported to bind ssDNA with a relatively low fluorescence signal [4]. The fluorescence of SGI and SYBR Gold displayed an ssDNA-concentration-dependent increase, as shown in Figure 1D and Figure 1E, respectively. Plotting the maximum fluorescence signal against the ssDNA concentration, both SGI and SYBR Gold exhibited a good linear relationship in the concentration range of 1 to 100 nM. The detection limit was calculated to be 0.6 nM with SGI and 0.3 nM with SYBR Gold, which was similar to that calculated for our CCP-based ssDNA biosensor.

### 3.2. The Influence of Salt on Our CCP-Based ssDNA-Binding Assay

**Figure 2:**
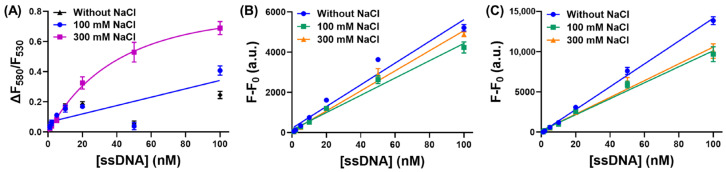
(**A**) The fluorescence ratio changes of PMNT in response to ssDNA concentration in the presence of 0–300 mM NaCl. The concentration of PMNT was 5 μM. The fluorescence intensity changes of SGI (**B**) and SYBR Gold (**C**) in response to ssDNA concentration in the presence of 0–300 mM NaCl. The concentration of SGI and SYBR Gold was 0.5×.

Since the main binding force between PMNT and ssDNA was electrostatic interactions, we first evaluated the influence of NaCl on our CCP-based ssDNA-binding assay. In the absence of NaCl, PMNT showed less pronounced fluorescence changes upon binding ssDNA. With 0.1M NaCl, the fluorescence ratio changes of PMNT were almost the same as those without NaCl, both of which were smaller than those with 0.3 M NaCl (Figure 2A). These results indicated that a higher salt concentration was more favorable for detection. Since too much salt would screen charge attraction and weaken the interactions between PMNT and ssDNA [31], we finally chose 0.3 M NaCl as the optimal salt concentration. In contrast, for the SGI and SYBR Gold-based staining method, the fluorescence changed only slightly with an increased concentration of NaCl (Figure 2B,C). In this regard, the DNA-staining dyes were more robust at various ionic strengths.

### 3.3. Effect of CCP Concentration

Under the optimized salt concentration, we further evaluated the concentration of PMNT. The most obvious red-shifted fluorescence should be related to the stoichiometric binding between PMNT and ssDNA. The random ssDNA we chose was a 24 nt sequence, which had 23 negative charges. With 1 μM PMNT, the fluorescence ratio saturated at 40 nM ssDNA, where the charge balanced, indicating that this method could quantitively detect ssDNA at less than 40 nM using 1 μM PMNT. Once the stoichiometric point was reached, the fluorescence ratio did not increase further in response to a higher ssDNA concentration. For 3 μM PMNT, the stoichiometric point was reached at an ssDNA concentration of about 100 nM, indicating that the maximum concentration for ssDNA measurement was lower than 100 nM using 3 μM PMNT. For 5 μM PMNT, the fluorescence ratio still increased at an ssDNA concentration of 100 nM, indicating a broader detection range. It is worth noting that the apparent *K*_d_ of each fluorescence ratio curve ranged from 7 nM to 57 nM (7 ± 2 nM for 1 μM PMNT, 31 ± 2 nM for 3 μM PMNT, and 57 ± 4 nM for 5 μM PMNT). Although they differed by several folds, these *K*_d_ values all represented an intimate binding affinity between ssDNA and PMNT. The lower *K*_d_ values were more likely to be close to the real *K*_d_, since the higher values might have been in the titration range due to the use of an excessively high probe concentration [37]. Considering both the *K*_d_ value and the detection range, we chose 5 μM PMNT for ssDNA detection.

### 3.4. Effect of pH

**Figure 3:**
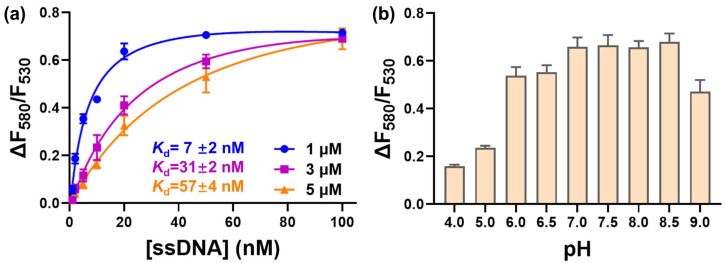
(**a**) The fluorescence ratio changes of PMNT in response to ssDNA concentration with PMNT ranging from 1 to 5 μM. (**b**) The fluorescence ratio changes of PMNT in response to different pH values. The concentration of ssDNA was 100 nM, and the concentration of PMNT was 5 μM.

The pH of the reaction buffer plays an important role during the binding between PMNT and ssDNA due to the possibility of DNA being (de)protonated at an extreme pH. We studied the effect of pH on the fluorescence changes of PMNT upon binding ssDNA. As depicted in Figure 3B, when the pH increased from 4 to 6, the fluorescence ratio changes also increased, and the fluorescence ratio changes reached their maximum at pH 7 to 8.5. After increasing the pH to 9, the fluorescence ratio changes showed a decreasing trend. Due to the specific structure of quaternary amine, the positive charges of PMNT were not changed between pH 4 and 9. On the other hand, DNA bases were more likely to be (de)protonated at a pH lower than 4 or higher than 9, which could destabilize ssDNA. Therefore, the optimal pH for PMNT binding with ssDNA was a neutral pH ranging from 6 to 8.5, and we finally chose 7.5 as the best pH.

### 3.5. Effect of DNA Sequence

**Figure 4:**
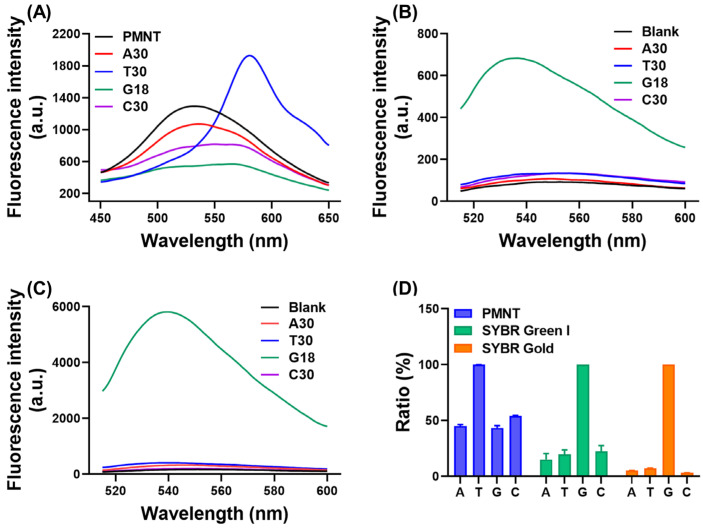
The fluorescence spectra of PMNT (**A**), SGI (**B**), and SYBR Gold (**C**) in response to different ssDNA sequences. (**D**) The ratio of other sequences compared with the highest signal. For the CCP-based method, the fluorescence ratio of poly-T was set as 100%. The intensity of poly-G was set as 100% for the SGI and SYBR Gold-based methods. The concentration of PMNT was 5 μM, and the concentration of SGI and SYBR Gold was 0.5×.

Different sequences of ssDNA may induce different fluorescence responses from PMNT depending on the base composition. Thus, we next assessed the effect of the sequence on the fluorescence changes of PMNT after binding with ssDNA. As depicted in Figure 4A, only decreased fluorescence at 530 nm was observed after binding with A30. For T30, G18, and C30, the fluorescence of PMNT red-shifted to 580 nm, with T30 showing the highest fluorescence at 580 nm. This may have been due to the fact that poly-T had weaker intra-strand interactions that could better stretch PMNT to a highly conjugated structure, resulting in a stronger fluorescence change.

Compared with the CCP-based method, the results were completely different for SGI and SYBR Gold (Figure 4B,C). The highest fluorescence signal was observed for poly-G. For the other sequences, the fluorescence signal was close to blank. The fluorescence signal of SGI after adding poly-G was significantly lower than that of SYBR Gold, since SGI was more suitable for dsDNA detection, its fluorescence after binding with ssDNA being at least 11-fold lower than with dsDNA [4].

To further assess the influence of the sequence on the different methods, we calculated the relative fluorescence signal by setting the highest signal as 100%. The results are shown in Figure 4D, and the signals of A30, G18, and C30 were almost 50% that of T30 according to our CCP-based method. On the other hand, the fluorescence of A30, T30, and C30 was under 20% that of G18 when using the SGI or SYBR Gold-based staining method, indicating a much stronger sequence effect.

### 3.6. Effect of dsDNA

**Figure 5:**
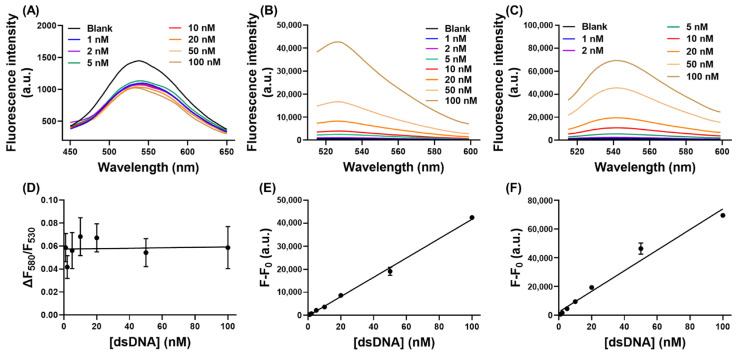
The fluorescence spectra of PMNT (**A**), SGI (**B**), and SYBR Gold (**C**) in response to different concentrations of dsDNA. (**D**) The fluorescence ratio of PMNT as a function of dsDNA concentration. The fluorescence intensity changes of SGI (**E**) and SYBR Gold (**F**) in response to dsDNA concentration. The concentration of PMNT was 5 μM, and the concentration of SGI and SYBR Gold was 0.5×.

As ssDNA may exist with a high concentration of dsDNA in the background, we further assessed the influence of dsDNA on our CCP-based ssDNA-binding assay. As shown in Figure 5A, the fluorescence spectra of PMNT barely changed upon binding with varied concentrations of dsDNA. Only slightly decreased fluorescence at 530 nm was observed without any red-shifted fluorescence at 580 nm. Thus, the fluorescence ratio of PMNT was almost unchanged with different concentrations of dsDNA (Figure 5D), indicating that our CCP-based ssDNA biosensor was not interfered with by background dsDNA. For the fluorescence-dye-based staining assay, the fluorescence intensity of both SGI and SYBR Gold showed a dsDNA-concentration-dependent increase, as depicted in Figure 5B and Figure 5C, respectively. Plotting the fluorescence intensity against the concentration of dsDNA, fine linear relationships were achieved for both SGI and SYBR Gold (Figure 5E,F). Therefore, our CCP-based ssDNA-binding assay was more suitable for the measurement of ssDNA in this regard.

### 3.7. Detection of ssDNA on AuNPs

To assess the practical application of our CCP-based method for the measurement of ssDNA, we studied the adsorption of both unlabeled and thiolated DNA on AuNPs. Firstly, unlabeled ssDNA was incubated with AuNPs and then washed three times. The precipitant was dissolved with KCN to destroy the AuNP cores in order to release the ssDNA. After mixing with the released ssDNA, the fluorescence ratio changes of PMNT were recorded to measure the adsorbed ssDNA concentration using the standard curve shown in Figure 1C. The concentration of released ssDNA was calculated to be 13.1 nM, with a binding efficiency of 56.6% and an average of 26 ssDNA strands absorbed on each AuNP (Table 1 and Table S2). To verify the accuracy of our CCP-based ssDNA-binding assay, 5′-FAM-labeled ssDNA was used in the adsorption experiment, and the fluorescence of the released ssDNA was then measured; we found that 59.1% of the FAM-labeled ssDNA was adsorbed on the AuNPs, which was similar to the results of our CCP-based ssDNA-binding assay.

After the quantification of the unlabeled ssDNA on AuNPs, we further applied our CCP-based ssDNA-binding assay to the measurement of thiolated ssDNA on SNA. We used 5′-SH-labeled ssDNA to form SNA via the freezing-directed method [34]. After removing the unbound DNA and adding KCN, the released ssDNA was mixed with PMNT, and the fluorescence of PMNT was detected. By plugging the fluorescence ratio into the standard curve (Figure S1), the concentration of the adsorbed ssDNA was calculated. The conjugation efficiency was determined as 47%, and an average of 130 ssDNA strands were absorbed on each AuNP (Table 1 and Table S2). The results were then verified by detecting the fluorescence of dual-labeled ssDNA (5′-SH-ssDNA-FAM-3′) after KCN dissolution using the standard curve obtained by detecting the fluorescence of FAM with different ssDNA concentrations (Figure S2) [38]. Similar results were obtained by detecting the fluorescence of fluorophore-labeled DNA, further verifying the accuracy of our CCP-based ssDNA-binding assay.

## 4. Discussion

Our CCP-based ssDNA-binding assay used a fluorescence ratio of 580 nm to 530 nm to quantify ssDNA, which could normalize the data differences caused by the slight changes in the initial fluorescence of PMNT. The use of a dual fluorescence signal guaranteed a detection accuracy superior to that of the single-fluorescence-peak detection method. The results were verified by the SGI and SYBR Gold-based staining methods. Although the CCPs SGI and SYBR Gold employ different mechanisms upon binding with ssDNA, they achieved a similar analytical performance, indicating the good practicability of our assay.

For reliable and quantitative analysis, the measurement of ssDNA should not be severely influenced by the sample sequences. However, for the fluorescence-dye-based method, the binding affinity of SGI and SYBR Gold with ssDNA was notably affected by the base composition. In contrast to the ssDNA containing poly-G, which showed extremely strong fluorescence, the ssDNA containing poly-A, poly-T, and poly-C was almost unstained (Figure 4B,C) [6], indicating that SGI and SYBR Gold were inappropriate for staining ssDNA in practical applications. For our CCP-based binding assay, the detection of ssDNA mainly relied on the conformational changes of PMNT caused by the stretching effect after binding with ssDNA. Although poly-T showed the highest fluorescence change due to the relatively weak intra-strand interactions, other sequences produced reasonable fluorescence changes, reaching almost 50% of that generated by poly-T. Apparently, our CCP-based binding assay showed higher tolerance toward different ssDNA sequences compared with the SGI and SYBR Gold-based staining methods, which ensures its widespread application.

Using fluorescence dyes to specifically detect ssDNA is susceptible to interference from dsDNA in the surrounding environment, especially for dsDNA-intercalating dyes. The fluorescence of SGI and SYBR Gold was dramatically enhanced with an increased concentration of dsDNA (Figure 5B,C). On the other hand, for the CCP-based ssDNA-binding assay, only slightly decreased PMNT fluorescence at 530 nm was observed, without any red-shifted fluorescence, and the fluorescence ratio of PMNT was almost unchanged by variations in the dsDNA concentration (Figure 5A,D). It is worth noting that this effect was not correlated with the dsDNA concentration, demonstrating that the existence of large amounts of dsDNA did not interfere with the specific detection of ssDNA. Therefore, our CCP-based ssDNA-binding assay was more suitable for the measurement of ssDNA compared with the fluorescence-dye-based methods. That being said, the DNA-staining dyes were more resistant to changes in the salt concentration, and they also showed a broader linear range.

## 5. Conclusions

In this work, we studied the interactions between CCPs and ssDNA based on the fluorescence changes of CCP after binding with ssDNA. ssDNA can stretch PMNT to a highly conjugated conformation with a decreased fluorescence at 530 nm or a red-shifted fluorescence at 580 nm. The optimized concentration of NaCl was 0.3 M. The fluorescence ratio of PMNT at 580 nm to 530 nm showed a good linear relationship with the ssDNA concentration, ranging from 1 to 20 nM. The detection limit for ssDNA detection was calculated to be 0.6 nM, which was similar to that determined for the SGI and SYBR Gold-based methods. Our CCP-based binding assay exhibited smaller signal differences when detecting ssDNA with different base components. Moreover, the existence of dsDNA did not interfere with the measurement of ssDNA, demonstrating that our CCP-based binding assay was more suitable for ssDNA detection compared with the fluorescence-dye-based methods. Further applying our CCP-based binding assay to the measurement of ssDNA on AuNPs, the accurate quantification of ssDNA on individual AuNPs was realized. Therefore, this assay could be applied for the detection of ssDNA on other surfaces, such as nanomaterials and microchips.

## Figures and Tables

**Table 1:** The binding efficiency of ssDNA on AuNPs.

DNA Type	Method	Binding Efficiency (3 Trials)			Average
Unlabeled DNA	CCP-based ssDNA-binding assay	56.0%	59.3%	54.4%	57 ± 3%
	Fluorescence-dye-based method	59.0%	59.1%	59.3%	59.1 ± 0.2%
Thiolated DNA	CCP-based ssDNA-binding assay	52.2%	46.4%	43.6%	47 ± 4%
	Fluorescence-dye-based method	46.6%	47.1%	47.3%	47.0 ± 0.4%

## Data Availability

Not applicable.

## Supplementary Materials

The following supporting information can be downloaded at: https://www.mdpi.com/article/10.3390/bios13010122/s1, Figure S1: (A) The fluorescence ratio of PMNT as a function of SH-ssDNA concentration. (B) The linear response at 1 to 20 nM SH-ssDNA, Figure S2: The fluorescence intensity changes of SH-ssDNA-FAM in response to ssDNA concentration, Table S1: The sequence of ssDNA used in this work, Table S2: The quantification of ssDNA on AuNPs.
