# Supplementary material for: A Label-Free, Mix-and-Detect ssDNA-Binding Assay Based on Cationic Conjugated Polymers
Source: Biosensors (Basel). 2023 Jan 10;13(1):122. doi: 10.3390/bios13010122 (PMC9855919; doi:10.3390/bios13010122)
Supplement: Supplementary file 1 [file biosensors-13-00122-s001.zip › biosensors-2085736-supplementary.pdf]

biosensors

MDPI

Supplementary Information

A Label-Free, Mix-and-Detect ssDNA-Binding Assay Based on Cationic Conjugated Polymers

Pengbo Zhang [1,2], Mohamad Zandieh [2], Yuzhe Ding [2], Lyuyuan Wu [2], Xiaoyu Wang [3], Juewen Liu [2,*] and Zhengping Li [1,*]

1 School of Chemistry and Biological Engineering, University of Science and Technology Beijing, 30 Xueyuan Road, Haidian District, Beijing 100083, China
2 Department of Chemistry, Waterloo Institute for Nanotechnology, University of Waterloo, 200 University Avenue West, Waterloo, ON N2L 3G1, Canada
3 School of Materials Science and Engineering, University of Science and Technology Beijing, 30 Xueyuan Road, Haidian District, Beijing 100083, China
* Correspondence: liujw@uwaterloo.ca (J.L.); lzpbd@ustb.edu.cn (Z.L.)

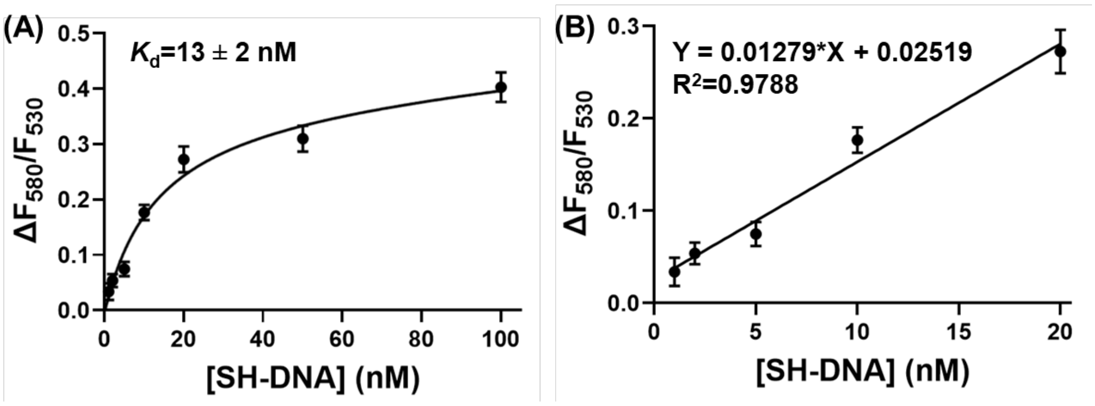

Figure S1. (**A**) The fluorescence ratio of PMNT as a function of SH-ssDNA concentration. (**B**) The linear response at 1 to 20 nM SH-ssDNA. The concentration of PMNT was 5 μM.

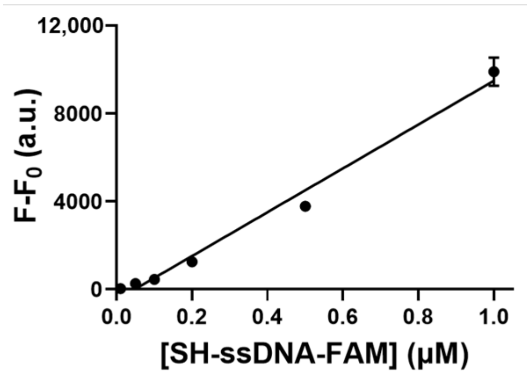

Figure S2. The fluorescence intensity changes of SH-ssDNA-FAM in response to ssDNA concentration.

Table S1. The sequences of ssDNA used in this work.

Names	Sequences (5'-3')
**ssDNA**	CCCAGGTTCTCTTCACAGATGCGT
**cDNA**	ACGCATCTGTGAAGAGAACCTGGG
**ssDNA-FAM**	CCCAGGTTCTCTTCACAGATGCGT-FAM
**SH-ssDNA**	SH-AAAAAAAAACCCAGGTTCTCT
**SH-ssDNA-FAM**	SH- AAAAAAAAACCCAGGTTCTCT-FAM
**A30**	AAAAAAAAAAAAAAAAAAAAAAAAAAAAAA
**T30**	TTTTTTTTTTTTTTTTTTTTTTTTTTTTTT
**G18**	GGGGGGGGGGGGGGGGGG
**C30**	CCCCCCCCCCCCCCCCCCCCCCCCCCCCCC

Table S2. The quantification of ssDNA on AuNPs using our CCP-based ssDNA binding assay.

DNA type	Supernatant	KCN released	Average ssDNA number on a single AuNP
Unlabeled DNA	39.9 nM	50.8 nM	26
	41.9 nM	61.0 nM	
	38.4 nM	45.7 nM	
Thiolated DNA	1.3 μM	1.4 μM	130
	1.5 μM	1.3 μM	
	1.5 μM	1.2 μM	
